# Patterns of Patients’ Interactions With a Health Care Organization and Their Impacts on Health Quality Measurements: Protocol for a Retrospective Cohort Study

**DOI:** 10.2196/10734

**Published:** 2018-11-07

**Authors:** Arriel Benis, Nissim Harel, Refael Barak Barkan, Einav Srulovici, Calanit Key

**Affiliations:** 1 Faculty of Technology Management Holon Institute of Technology Holon Israel; 2 Clalit Research Institute Clalit Health Services Tel-Aviv Israel; 3 Department of Computer Sciences Faculty of Sciences HIT - Holon Institute of Technology Holon Israel; 4 School of Nursing University of Haifa Haifa Israel; 5 Clalit Community Division Clalit Health Services Tel-Aviv Israel

**Keywords:** health communication, population characteristics, eHealth, mHealth, telehealth, health information systems, consumer health informatics, delivery of health care, machine learning

## Abstract

**Background:**

Data collected by health care organizations consist of medical information and documentation of interactions with patients through different communication channels. This enables the health care organization to measure various features of its performance such as activity, efficiency, adherence to a treatment, and different quality indicators. This information can be linked to sociodemographic, clinical, and communication data with the health care providers and administrative teams. Analyzing all these measurements together may provide insights into the different types of patient behaviors or more accurately to the different types of interactions patients have with the health care organizations.

**Objective:**

The primary aim of this study is to characterize usage profiles of the available communication channels with the health care organization. The main objective is to suggest new ways to encourage the usage of the most appropriate communication channel based on the patient’s profile. The first hypothesis is that the patient’s follow-up and clinical outcomes are influenced by the patient’s preferred communication channels with the health care organization. The second hypothesis is that the adoption of newly introduced communication channels between the patient and the health care organization is influenced by the patient’s sociodemographic or clinical profile. The third hypothesis is that the introduction of a new communication channel influences the usage of existing communication channels.

**Methods:**

All relevant data will be extracted from the Clalit Health Services data warehouse, the largest health care management organization in Israel. Data analysis process will use data mining approach as a process of discovering new knowledge and dealing with processing data extracted with statistical methods, machine learning algorithms, and information visualization tools. More specifically, we will mainly use the k-means clustering algorithm for discretization purposes and patients’ profile building, a hierarchical clustering algorithm, and heat maps for generating a visualization of the different communication profiles. In addition, patients’ interviews will be conducted to complement the information drawn from the data analysis phase with the aim of suggesting ways to optimize existing communication flows.

**Results:**

The project was funded in 2016. Data analysis is currently under way and the results are expected to be submitted for publication in 2019. Identification of patient profiles will allow the health care organization to improve its accessibility to patients and their engagement, which in turn will achieve a better treatment adherence, quality of care, and patient experience.

**Conclusions:**

Defining solutions to increase patient accessibility to health care organization by matching the communication channels to the patient’s profile and to change the health care organization’s communication with the patient to a highly proactive one will increase the patient’s engagement according to his or her profile.

**International Registered Report Identifier (IRRID):**

RR1-10.2196/10734

## Introduction

### Background

Health care organizations and patients communicate with each other using various communication channels [1,2]. Some of these communication channels are traditional: face-to-face meetings with a physician or a nurse, face-to-face interactions with the administrative staff, and phone calls. However, in the past decade, many health care organizations introduced novel methods of digital communication with patients such as text messages, emails, video calls, websites, and mobile apps. The communication channels between the health care organization and its patients have been examined and analyzed in previous studies [3-10].

Data mining and machine learning methodologies have been used to define or redefine clusters of patients according to their state of health and other sociodemographic data [11,12]. Recently, process mining has been used to try to improve communication between consumers and health care providers [13]. However, no studies attempting to cluster patients by combining medical, sociodemographic, or communication characteristics have been conducted and certainly not in a population as large as the one proposed in this study. We expect that such research will improve communication between patients, service providers, and medical organizations and will improve the quality of treatment and treatment effectiveness and responsiveness.

### Aims and Objectives

Finding the circumstances and the extent to which different population segments use different communication channels, and specifically, the extent to which usage of newly introduced channels replaces the usage of more traditional channels will help us learn about the effectiveness of these new channels. Tying these population segments’ communication behavior with their sociodemographic profiles and health outcomes will help us establish the association between the 3, and it may help drive the hypotheses as to the causation. In addition, identifying communication-based population segments may help health care providers to use the most appropriate channels with each population segment, leading to more efficient and targeted communications, for example, identifying and quantifying the early adopters group will help the health care organization to estimate the usage level of a newly developed communication channel, its effectiveness in driving the intended message, and to some extent, its effect on health outcomes. Accordingly, this will also allow to improve the quality of treatment, treatment effectiveness, and responsiveness.

The aims of this retrospective data study are to assist health care policy makers to improve and personalize the communication between patients and health care professionals (eg, physicians and nurses). Communication improvement includes enhancing the accessibility of health care professionals by expanding the capabilities of current communication channels and introducing new ones. These communications will help to improve patient engagement with the treatment process, increase patient responsiveness to follow-up requirements and treatment, and improve patient experience with health care services. More specifically, the primary aim of this study is to characterize usage profiles in the available communication channels in the Clalit Health Services (Clalit), each one of them without considering the others and then all of them together. The second aim is to establish relationships between communication profiles, sociodemographic, and medical patients’ profiles. The main objective is to suggest new ways to encourage the usage of the most appropriate communication channel based on the patient’s profile. A secondary objective is to suggest ways for improving communication between the patient and the health care organization mainly through technological means.

### Hypotheses

The first hypothesis is that the patient’s follow-up and clinical outcomes are influenced by the patient’s preferred channel(s) of communication with the health care organization. If this hypothesis is validated, the research will quantify the phenomenon.

The second hypothesis is that the adoption of newly introduced communication channels between the patient and the health care organization is influenced by the patient’s sociodemographic and/or clinical profile. If this hypothesis is validated, the research will identify sociodemographic and/or clinical attributes that affect the adoption of newly introduced communication channels.

The third hypothesis is that the introduction of a new communication channel influences the usage of existing communication channels. If this hypothesis is validated, the research will characterize the changes in usage of existing communication channels once a new communication channel is introduced.

## Methods

### Materials

This is a data-based study that analyzes information stored in Clalit electronic medical records (EMRs) and in logs documenting access to various communication channels between patients and Clalit, such as the internet personal health records, and telephone logs. Researchers have full access to Clalit EMRs and logs on the entire insured population of 4.53 million patients in 2015, which constitute 54% of the Israeli population of 8.38 million as of 2015. Data collected include demographic, clinical, and pharmacological information. In addition, we plan to conduct interviews with a representative sample of the patients to learn directly about the patients’ perceptions, their relationship with the various means of communication, patterns of use, and suggestions for improvement. We hope that this survey will provide supplementary information to the one we will receive from analyzing the data.

Clinical data from community and hospital settings and pharmacological data are routinely collected in the data warehouses (DWHs) of the health maintenance organization (HMO) and classified into the appropriate data world (eg, appointment scheduling, consultation with a physician, appointment with a specialist, diagnosis during hospitalization, medical services, and prescriptions). The information recorded includes sociodemographic data (gender, marital status, number of children at home, age, origin, socioeconomic status (SES), and place of residence), medical information (dates of specialist appointment, physician license number and the corresponding specialization, diagnoses, date of each diagnosis, prescriptions, acquisition of prescriptions, laboratory results, and imaging), and communication data (appointment date, date the appointment occurred, time elapsed between the scheduled appointment and the actual appointment, and the way the appointment was scheduled—through a medical secretary, call center, website, or mobile app). All relevant pieces of information include a patient identifier, which allows compiling all data relevant to a specific patient into a single record.

The information to be analyzed is extracted from the EHR DWH of Clalit and includes data collected between 2008 and 2016 for all relevant patients. The long duration of the study will allow us to identify changes in the ways patients interact with the HMO as a function of time and as a function of new communication channels the HMO introduced (eg, website, mobile apps, and the use of the short message service [SMS] text messaging). Accordingly, the patient can start or stop using 1 or more channels to interact with the HMO. The patients included in this study are aged 21 years and over and are members of Clalit for at least 1 year before 2008 and are still alive in 2016. We will focus our study on patients with chronic disease because we want to examine long-term adherence and efficacy. In addition, patients who suffer from 1 chronic disease or more have a high rate of resource consumption. In the United States, for example, 86% of health care spending is devoted to patients with chronic diseases [14]. In particular, we will examine diabetic patients, who in 2001 accounted for about 20% of the patient population [15]. We hope that the study will help optimize the processes in which these patients participate. The incidence of chronic diseases in general and of diabetes in particular is increasing over the years due to several factors, most notably the aging of the Israeli population. According to Clalit data, as of the end of 2014, more than 40% of the insured population had at least 1 diagnosis that is defined as chronic (eg, diabetes, asthma, heart disease, mental illness, and cancer). Patients with diabetes constitute more than 300,000 individuals with our inclusion criteria [16,17]. The profiles that will be found will help define the recommendations and policies that will improve communication with specific subpopulation groups and will increase the effectiveness of treatment and patient adherence. Chronic diseases are not spread uniformly by age; however, given the high cost of treating patients with chronic diseases, we believe it is more useful to concentrate on these patients despite this bias.

### Ethics

Ethical approval for the study was granted by the Clalit ethical committee (147-15-COM2; January 26, 2016).

### Methodologies

The communication between health care providers (ie, physicians, nurses, hospitals, and more globally, HMOs) and patients is studied by focusing, generally, only on 1 or 2 of the channels [1-12]. To fulfill our research aims and objectives, our analysis will consist of characterizing the usage profiles of existing nontechnological and technological communication channels over a period of 9 years, taking into account that Clalit has added and changed over the time the methods by which patients contact health care professionals (eg, the introduction of Web and mobile apps). Then, the sociodemographic and clinical profiles of each one of the different communication channels’ usage profiles will be defined. This will allow us to qualitatively evaluate the influence of the communication profile on patient’s engagement and follow-up quality.

As part of the analysis, we will evaluate impacts of new communication channels introduced over the research period. This will allow us to suggest future improvements to the communication between the patient and physician or nurse, with the aim of improving the work processes of the health organization.

This research is based on knowledge discovery in databases (KDD) methodologies [18,19]. KDD is an interdisciplinary discipline that deals with methodologies for the extraction and identification of valid, new, nontrivial patterns of data that have the potential to be useful and understandable [18-20]. The continued increase in the amounts of data available, a product of the unprecedented development of computer and communications technologies over the past two decades, created a unique opportunity to implement KDD methodologies. Data science experts from different disciplines are therefore challenged to find new and effective ways to extract and generate new knowledge from existing data.

In the analysis phase, we will use one-dimensional and multidimensional statistical methods as well as different data mining algorithms. The data mining stage is part of the KDD process and focuses mainly on the discovery of unknown patterns. For this purpose, we will use and tune, if necessary, data mining [21] and machine learning [22] algorithms for dealing with the multidimensional dataset (ie, sociodemographics, bio-clinical, and communication-related data over time), which will be explored in this study. The patterns found in this stage are then evaluated and interpreted to form the knowledge extracted from the KDD process.

The KDD process that will be developed and implemented in this research includes data collection and integration, early processing and cleaning of data, development and implementation of data mining algorithms to discover new knowledge and a qualitative research [18-20].

#### Data Acquisition

Clalit DWH is the main source of information the research uses, and a replication for research purposes is updated on a weekly basis. The data extracted from Clalit DWH for each patient comprise the following information:

1. Sociodemographic data
  - Date and country of birth and date of immigration when relevant
  - Date of death (allowing exclusion)
  - Start and end date of membership (allowing exclusion)
  - Gender
  - Ethnic sector (general Jewish, Arab, and ultra-orthodox Jewish)—the ethnic sector is determined according to the clinic at which the member receives primary care medicine. It is computed by the Clalit computer services unit by integrating geostatistical data from the Israeli Central Bureau of Statistics
  - Clinic-level SES (3 categories: low, mid, and high)—the SES is determined according to the clinic at which the member receives primary care medicine. It is computed by the Clalit computer services unit by integrating geostatistical data from the Israeli Central Bureau of Statistics
2. Bio-clinical
  - Body mass index (BMI) category (underweight, normal, overweight, obese, or unknown) [23]
  - Smoking status (current, past, never, or unknown)
  - Last available glycated hemoglobin (HbA_1c_) measurement reflecting the level of blood sugar control in patient with diabetes
  - Last available lipidemic profiling (high-density lipoprotein, low-density lipoprotein, triglycerides, and total cholesterol)
  - Adjusted clinical groups (ACG) [24]
  - Comorbidities according to the Clalit chronic diseases registry [15]
  - Proportion of days covered by treatment of diabetes when relevant based on purchase of drugs used in diabetes and more particularly by blood glucose lowering drugs excluding insulin (Anatomical Therapeutic Chemical Classification System codes starting with A10B) [25]
3. Communication or contacts with the HMO data
  - Appointments scheduling (through a medical secretary—data available since 2009, call center—data available since 2009, website—since 2011, or mobile app—since 2012)
  - Consultations with a physician or a nurse
  - Hospitalizations
  - Consultations at an emergency department
  - Nonqueue requests (eg, request for periodic checks, prescription renewal, and sick leave certificate) done without visiting but only by sending a request to a physician through a call to a medical secretary or a nurse or by completing a paper or an electronic form
  - Any purchases in a pharmacy of the HMO or purchase related to a prescription in other pharmacies having an agreement with the HMO
  - Prescription renewals by SMS—since 2015.

#### Data Preprocessing

##### Data Cleansing

After integrating the data collected and extracted from the Clalit’s DWH, we will prepare it for analysis. This stage includes cleansing of the data collected by Clalit’s DWH when necessary. The main objective of this phase is to reduce noise by detecting and removing or correcting outliers [26] in the dataset by evaluating the quality of the data [21]. An outlier is a data measurement that is inconsistent with other historical measurement data of the same individual (eg, outlaying height value, an exceptionally high number of consultations with a physician—a few hundred per year-). When a measurement-specific (eg, BMI) algorithm has been developed in-house by Clalit Research Institute (CRI) for epidemiological studies, outlier detection and data correction will be processed using it. For example, an algorithm screens data on BMI, weight, and height, to detect and handle outliers in the recording of 1 of these 3 measurements (eg, due to mistyping). When the CRI algorithms will not be relevant, outliers will be detected with statistical approaches such as median absolute deviation to find outliers (nonparametric due to lack of knowledge regarding the data distribution [27] and/or machine learning algorithms such as k-means [28]).

Data related to communication between patient and Clalit have not yet been fully processed and cleansed before, and accordingly, we may need to develop special cleaning and correction algorithms for these data. If data correction algorithms and/or algorithms that deal with cases of missing information do not exist for any given data in our database [29,30], we will use appropriate machine learning algorithms and/or statistical approaches [31,32] to correct and/or deal with missing data where needed. Examples of potential problems that we might encounter are identifying irrelevant entries (eg, entries related to quality assurance traffic and testing and entries that are not the result of human activity) and lack of full documentation. In addition, interface exposed to the user is a *breathing* interface and changes over time depending on the services that the HMO chooses to provide through the Web-based and app services. A new version of the website, for example, is released every 6 months. Data processing and analysis should reflect these changes.

##### Data Transformation

Many methods of machine learning and data mining require, as part of the preprocessing phase, a data reformulation such as a new categorization or a new grouping of numerical, categorical, or textual data to reduce the number of values each attribute has [28].

This step involves the use of techniques for reducing the number of dimensions or transduction methods to reduce the number of variables for analysis or to find invariant representations of the data [26,33-35].

For example, if we consider attributes with continuous values such as laboratory tests or clinical measurement having existing and defined scales in the literature, we will reformulate them into categorical values as a part of the dataset dimension reduction. For example, HbA_1c_ values may be divided into 5 categories: excellent control (<6.5%), good control (6.5% to 7.5%), moderate control (7.5% to 8.9%), poor control (**≥**9%), and not available [36,16].

However, for attributes that do not have predefined scales in the literature or which are specific to Clalit, such as the number of appointments by using the HMO website or the number of visits to a physician per year, we will use the k-means clustering algorithm for discretization purposes in 6 groups of resource consumption: “No” (meaning not consuming of the related resource, so excluded from the k-means run and assigned to this group), “Small,” “Small-Moderate,” “Moderate,” “Moderate-Large,” and “Large.” The cluster bounds are validated, if necessary, by a domain expert (ie, a public health practitioner having some experience with the Clalit data).

#### Data Mining

For identifying population clusters, different machine learning methods and algorithms must be used. The main aim is to characterize usage profiles in the available communication channels. Considering the fact that we do not have prior knowledge on the data, we will use unsupervised machine learning algorithms [37-43] and will more particularly focus on k-means [38] and hierarchical clustering [37]. We choose to use these specific algorithms because they are relatively simple to communicate with people having less technical knowledge, such as decision and policy makers of the HMO, which will get the final analysis report and will need to implement its recommendations.

The first data mining goal is to find the number of hidden k clusters in the “Communication/contacts with the HMO data” or in other words, the number of different types of patient communication profiles. This will be performed on the available data of the year 2016 because by that time, data cleansing will be fully performed. As communication channels constantly evolve, we chose the most recent year to be the reference point to which previous years, with less communication channels, are compared with. The “Communication/contacts with the HMO data” of 2016 will be clustered as follows:

1. For each k between 2 and 100, 100 randomly selected samples of 20% of the cohort will be generated
2. For each sample, k-means will be run
3. For each run, the Ray-Turi criterion [44] will be computed
4. The results of the overall Ray-Turi criterion computation will be plotted on a graph
5. The elbow will be manually defined on the previously built plot for finding the relevant k.

Each cluster relates to a type of patient communication. This step allows reducing the patient communication profiles from the number of patients included into the cohort (more than 300,000 if we consider patients with diabetes) to a small one (at most less than a few dozen).

The second data mining goal is to generate a hierarchical clustering of the previously discovered clusters to allow understanding the similarities and dissimilarities between the communication patterns.

Descriptive statistics of sociodemographic, bio-medical, and communication data will be generated for each cluster.

On the basis of the previously built k clusters of “Communication/contacts with the HMO data” of 2016 and the related hierarchical clustering, we will generate descriptive statistics for each patient communication profiles (ie, cluster or set of patients) over the years (2008-2015).

#### Information Visualization

To provide user-friendly tools to decision and policy makers [45], allowing them to understand the different patient communication profiles and the strengths and weaknesses of each one, we will build heat maps for each year between 2008 and 2016 based on the previously generated hierarchical clustering of 2016 data.

#### Process Mining

Furthermore, we plan to implement algorithms and approaches from the field of process mining [46] to identify the changes in communication profiles over time, which may be the cause of treatment adherence changes. For example, process mining will allow us to model how patients with a similar communication profile (ie, patients within the same cluster) have changed their communication patterns with the HMO using the following channels:

1. Consulting with physicians and/or nurses
2. Scheduling appointments by using 1 or more of the following channels: through a medical secretary—data available since 2009, call center—data available since 2009, website—since 2011, or mobile app—since 2012
3. Overall interaction with the HMO (using the overall services).

#### Qualitative Research

Qualitative research of focus groups is the most effective means to fully understand factors that encourage or delay the use of communication interfaces with the health care organization. Focus groups enable the collection of information from a multicultural population [47] and discussion of new ideas that do not arise during personal interviews [48]. We designed the qualitative part of the proposed study based on the guidelines presented by King et al [49]. The qualitative part of the research will include between 1 and 8 focus groups depending on their usage level of the communications channels with Clalit. Each one of the focus groups will include up to 8 patients from the same area. Participants in the focus groups will be asked to complete a short sociodemographic questionnaire and sign an informed consent form. During the focus group meeting, the group facilitator will record the discussion and make important notes related to the participants’ nonverbal communication.

A guideline questionnaire for the focus groups will be constructed with the assistance of experts in the field and relevant literature. This questionnaire will evaluate factors that encourage or delay the use of communication channels with Clalit. The guiding questionnaire will include up to 10 open questions that will facilitate responses providing critical information, for example, “What factors contribute or will contribute to your use of the communication channel X?”; “What factors delay or will delay your usage of communication channel X?”; or “How do you think that communication channel X can be improved?”. The guiding questionnaire will be used to explore aspects that are relevant for better understanding the topic and will facilitate expanding the discussion to areas that the participants consider to be most significant.

The discussions in the focus groups will be recorded and transcribed. The transcripts of the focus group discussion will be analyzed in a phenomenological approach that emphasizes the patient’s unique and subjective perception through qualitative content analysis [50]. The coding process will begin with open coding (ie, identification of major categories), following by axial coding that results from 1 core phenomenon. Next, the data will be categorized according to this core phenomenon [51] and will be reviewed by external domain experts to ensure objectivity [49]. Sandelowski [52] notes that through qualitative content analysis, researchers can add new information to the existing one and gain new insights. The encoding and analysis will be performed by the principal investigators and the associate investigators, with the same encoding rules for guaranteeing homogeneous and consistent encoding [49]. In cases of disagreement regarding the encoding, an expanded forum will be held in which the majority decision prevails.

## Results

This project was funded in 2016, and the research project is scheduled to be completed in 2019.

A preliminary analysis has been performed on the data of the year 2015 related to 309,460 patients with diabetes in 2015, aged 32 years and above, having the disease treated by Clalit for more than 7 years. Overall, 7 main communication patterns have been discovered.

The first cluster is of patients with relatively low contacts with the HMO in comparison with the rest of the population. Patients in these 2 groups tend to be relatively young (median age: 64 years) and less morbid (ACG between 3 and 4). Although patients in the first group tend to have a poor follow-up quality, 21.21% (18,779/88,524) of the patients were missing BMI measurement and 23.09% (20,436/88,524) were missing their HbA_1c_ measurement in 2015; patients in the second cluster have an average follow-up quality: only 7.72% (6228/80,714) of the patients did not perform a BMI measurement and only 10.56% (8527/80,714) did not perform a HbA_1c_ measurement. A possible explanation for this difference may be related to the tendency of the patients in the second group to resort mainly to human contact (face-to-face or by phone).

The next 2 clusters are of early adopters of technology. These diabetic patients interacted in 2015 with Clalit mainly through new digital platforms: the website (first group) or the mobile app (the second group). These patients also tend to use lesser medical services compared with the rest of the population, and their follow-up quality was better than the rest of the population: only 4.64% (1212/26,098) and 6.10% (1593/26,098) of the first group did not perform BMI and HbA_1c_ tests in 2015, respectively, whereas 5.05% (603/11,945) and 6.93% (826/11,945) of the second group did not perform BMI and HbA_1c_ tests in 2015, respectively.

The patients included in the fifth cluster are mainly using nursing services. They also tend not to schedule appointments. This subpopulation has a low SES (40.79%, 14,531/35,624). However, the follow-up of these patients is quite good (with 3.17% [1128/35,624] and 6.05% [2155/35,624] of these patients missing their BMI and HbA_1c_ measurements, respectively). This is a clear effect of the nursing personnel involvement.

Patients in the last 2 clusters tend to be older than the rest of the patient population (aged more than 70 years) and with relatively high morbidity (ACG=5). Patients in the sixth cluster tend to be consumers of medical services that involve access to a human being, whereas patients in the seventh cluster tend to be heavy users of all medical services. They also tend to have one of the best follow-up rates: only 1.64% (825/38,070) and 4.38% (1668/38,070) of the patients in the sixth cluster have missed their BMI and HbA_1c_ measurements, respectively, in 2015, whereas only 4.22% (1203/28,485) and 6.40% (1822/28,485) of the patients in the seventh cluster have missed their BMI and HbA_1c_ measurements, respectively, in 2015.

## Discussion

### Overview

This research protocol deals with the identification of patient communication profiles. This knowledge will help the health care organization to increase the accessibility of patients to the services the health care organization provides and to improve patients’ engagement with the treatment process. This, in turn, may motivate the patient to achieve a better treatment adherence, improve quality of care, and generate better patient experience.

### Expected Results and Future Directions

Analysis of communication patterns over time may reveal long-term behavior patterns as well as identify patterns at a higher abstraction level (eg, early adopters of technology and early adopters of services). It should be noted that the research is planned to be performed on data from a period that witnessed a significant yet gradual change in the communication channels Clalit provides its patients. Analyzing the response of the patient population to these changes will hopefully help improve the available communication channels as well as assist in formulating realistic expectations from the introduction of new communication channels, taking into consideration also the sociodemographic characteristics and clinical constraints as well as their previous communication patterns with the HMO.

By tuning its communication tools to patients’ preferences (eg, by translating the user interfaces of the electronic communications tools—website or apps—from Hebrew to other languages such as Arabic, English, Russian, Amharic, French, and Spanish), the health organization would (1) improve and increase accessibility to health care services, achieve better patient engagement and responsiveness to treatment, and improve quality of treatment and treatment experience within existing budgetary constraints and (2) increase patients’ engagement with the treatment process by transforming the communication scheme with each patient to a more proactive scheme, so as to better fit their profile.

### Strengths and Limitations

Clalit insured and provided medical services to approximately 4.53 million patients in 2015 and is the largest health care provider in Israel. The data available spans all treatment providers including hospitals’ end emergency units. Nevertheless, overall ethnic distribution of the Clalit population does not fully reflect the overall Israeli demographic composition. The Clalit members comprise, in comparison with the Israeli general population, (1) a higher proportion of Arabs and a lower proportion of ultra-orthodox members and (2) a higher proportion of members having a low SES.

Another potential limitation is the decision to analyze only patients with diabetes. These patients may exhibit behaviors that are unique to this specific chronic disease and may not be shared by other chronic patients. Nevertheless, diabetes is 1 of the most common chronic diseases, with prevalence of approximately 7% within Clalit’s insured population.

Finally, this research is conducted on data of Israeli patients. The structure of the Israeli health care system as well as Israeli culture and norms may affect patients’ behavior and may not apply to patients in other geographical locations.

## Abbreviations

ACG: adjusted clinical groups
BMI: body mass index
Clalit: Clalit Health Services
CRI: Clalit Research Institute
DWH: data warehouse
EMR: electronic medical record
HMO: health maintenance organization
HbA _1c_: glycated hemoglobin
KDD: knowledge discovery in databases
SES: socioeconomic status
SMS: short message service
